# School Climate, Loneliness, and Problematic Online Game Use Among Chinese Adolescents: The Moderating Effect of Intentional Self-Regulation

**DOI:** 10.3389/fpubh.2019.00090

**Published:** 2019-04-30

**Authors:** Chengfu Yu, Wentao Li, Qiao Liang, Xuelan Liu, Wei Zhang, Hong Lu, Kai Dou, Xiaodong Xie, Xiong Gan

**Affiliations:** ^1^School of Education, Guangzhou University, Guangzhou, China; ^2^School of Education, Center for Brain and Cognitive Sciences, Guangzhou University, Guangzhou, China; ^3^School of Psychology, South China Normal University, Guangzhou, China; ^4^Human Resources Department, South China Normal University, Guangzhou, China; ^5^School of Education, Yangtze University, Jingzhou, China

**Keywords:** adolescent, school climate, problematic online game use (POGU), loneliness, intentional self-regulation

## Abstract

Evidently, the school climate is important in reducing adolescent problematic online game use (POGU); however, the mechanism accounting for this association remains largely unknown. This study examined whether loneliness mediated the link between school climate and adolescent POGU and whether this mediating process was moderated by adolescent intentional self-regulation. To this end, self-report questionnaires were distributed. Participants were 500 12–17-years-old Chinese adolescents (*Mean*_age_ = 13.59 years, 50.60% male). After controlling for adolescents' gender, age, family socioeconomic status, and self-esteem, the results showed that the negative association between school climate and adolescent POGU was partially mediated by loneliness. Moreover, this indirect link was stronger for adolescents with low intentional self-regulation than for those with high intentional self-regulation. These findings highlight loneliness as a potential mechanism linking school climate to adolescent POGU and provide guidance for the development of effective interventions for addressing the adverse effects of a negative school climate.

## Introduction

Over the past two decades, problematic online game use (POGU) as a global public health issue has received increasing research interest (1–3). POGU, a subtype of problematic Internet use, refers to the uncontrollable, excessive, and compulsive use of online games that causes social and/or emotional problems (4). Individuals with POGU spend more time gaming than planned at the expense of other important activities, causing negative social and academic outcomes. Increasing evidence has consistently confirmed that POGU is associated with a variety of negative outcomes such as poor academic performance, depression, and aggression (3, 5, 6). Specifically, China has one of the highest adolescent POGU prevalence rates in the world raging between 2.2 and 21.5% (1, 7, 8). Therefore, an investigation of the factors that predict POGU is urgently needed to support the development of intervention programs.

Given that adolescents spend an increasing amount of their time engaged in school-related tasks, the influence of school contexts on adolescent development has received increased attention in the past decade (9–11). School climate refers to all relationships that affect children's cognitive, social, and psychological development, including adult-adult, adult-student, student-student, family-school, and community-school relationships (9, 12). However, perceptions of specific school climate may vary greatly across individuals.

According to the stage-environment fit theory (13, 14), optimal development takes place when school contexts adequately satisfy adolescents' increasing psychological needs for autonomy, relatedness, and competence. In this study, we primarily focused on three components of school climate: teacher support, student-student support, and opportunities for autonomy at school. Particularly, teacher support and student-student support may help meet adolescents' relatedness and competence needs. Moreover, teacher autonomy support can help to satisfy students' needs for autonomy, as well as offer students the opportunity to achieve competence and establish positive interactions with teachers and peers (15, 16). There is considerable evidence suggesting that students' perceptions of relatedness and autonomy in the school setting influence adolescents' academic adjustment as well as their physical and socio-emotional well-being (9, 17, 18). By the same token, a mismatch between school climate and the three aforementioned psychological needs can result in problem behaviors such as POGU.

Research has indicated that adolescents who perceive the school climate as favorable are less likely to develop POGU (10, 11, 19). For instance, Rehbein and Baier (11) found that students' perceptions of favorable school climates were an important protective factor against POGU in a 5-year longitudinal study of 406 students in grades 4–9. Similarly, Yu et al. (19) reported that 7th grade adolescents who perceived opportunities for autonomy at school had a decreased incidence of 9th grade POGU; this association was mediated via increased 8th grade basic psychological needs satisfaction and 9th grade school engagement. These findings highlight the merit of favorable school climate in reducing adolescent POGU.

## Loneliness as a Mediator

Although the association between school climate and adolescent POGU has been well-established, the mediating and moderating mechanisms underlying this relation are still under-investigated. Loneliness is prevalent in adolescents (20). According to the self-system processes model (21), a favorable school climate helps to reduce the degree of loneliness experienced by adolescents, which in turn reduces the risk of problem behaviors. In other words, loneliness may be an important mediator of the link between school climate and adolescent problem behaviors. When adolescents' socio-emotional needs are not adequately met by contextual factors such as school climate, the feeling of loneliness occurs (22). Moreover, adolescents suffering from loneliness are at elevated risk for POGU (5).

From one perspective, a school climate that responds to adolescent psychological needs for relatedness, competence, and autonomy, renders adolescents less likely to experience loneliness. Ample research evidence has confirmed the negative association between a favorable school climate and loneliness (23–25). For instance, Benner (23) reported that a positive school climate was negatively associated with loneliness. Similarly, Liu et al. (24) and Yu (25) found that junior middle school students who had more favorable school climate perceptions (positive teacher-student support, student-student support, and opportunities for autonomy at school) were less likely to experience loneliness.

From another perspective, when adolescents experience loneliness, they are more likely to indulge in online games. Growing numbers of studies support the important effect of loneliness in shaping adolescent POGU (5, 6, 26). For instance, Caplan et al. (26) found that loneliness was positively associated with POGU. Similarly, Qin (27) found that loneliness was a risk factor for POGU. In addition, Lemmens et al. (6) reported that loneliness was a significant and powerful predictor of POGU. Moreover, Chen and Fu (5) found that adolescents with POGU scored significantly higher on measures of loneliness than did adolescents without POGU. Taken together, these data led to the following hypothesis:

***Hypothesis 1:*** Loneliness will mediate the relationship between school climate and adolescent POGU.

## Intentional Self-Regulation as a Moderator

Despite that a robust relationship between school climate and adolescent POGU has been suggested in previous research, not all adolescents who experience a negative school climate develop POGU; some adolescents still adapt well even though they have negative perceptions of their school climate. Similarly, some adolescents still experience maladjustment even though they perceive their school climate favorably. Such variability in adolescents' responses to the school environment suggests that individual characteristics may play a key role in this observed heterogeneity.

According to the ecological system theory Bronfenbrenner (28), adolescents' development stems from the interplay between important contexts (such as school climate) and their intrapersonal characteristics. Among the many intrapersonal characteristics influencing adolescents' emotional problems (such as loneliness) and deviant behaviors, intentional self-regulation is an important moderator (29–31). Intentional self-regulation refers to one's efficiency in examining his/her abilities and negotiating his/her resources in the context of personal goals in order to attain better functioning and to enhance self-development (32, 33). Consequently, appropriate goals and goal-related strategies for attaining positive individual-context relations should be chosen (34). Thus, people with different levels of intentional self-regulation are influenced by contextual factors differently. More precisely, adolescents with good intentional self-regulation are more inclined to select suitable goals, optimize their own resources, and/or actively search for alternatives when failure happens, in turn increasing adjustment and reducing problem behaviors such as POGU (30, 32).

The risk-buffering hypothesis proposes that favorable personal characteristics such as intentional self-regulation can weaken the link between environmental stress (such as negative school climate) and problem behaviors (35). Consistent with this hypothesis, Urban et al. (31) found that intentional self-regulation moderated the relationship between neighborhood contexts and adolescent mental health symptoms, such that neighborhood risk factors were associated with increased mental health symptoms including loneliness, depression, and sadness among individuals with lower intentional self-regulation, but not among those with higher intentional self-regulation. This could be because adolescents with higher intentional self-regulation can obtain more coping resources from their neighborhood contexts. Similarly, adolescents with high intentional self-regulation tend to have clear goals and a vision for what they want to achieve, thus they can make good use of school resources and undergo more optimal development (such as less loneliness). Further, when faced with an unfavorable school climate, adolescents with high intentional self-regulation might be better able to focus on their goals and plans, thus reducing their sense of loneliness. Although they may experience setbacks and negative feelings (such as loneliness) when in a disadvantageous school climate, adolescents with excellent intentional self-regulation can adjust better and recover more quickly than those with poorer intentional self-regulation. Therefore, we proposed the following hypothesis:

***Hypothesis 2:*** Intentional self-regulation will moderate the indirect link between school climate and adolescent POGU. Specifically, the indirect association between school climate and POGU via loneliness will be stronger among adolescents with low-level intentional self-regulation and weaker among adolescents with high-level intentional self-regulation.

## The Present Study

**Figure 1:**
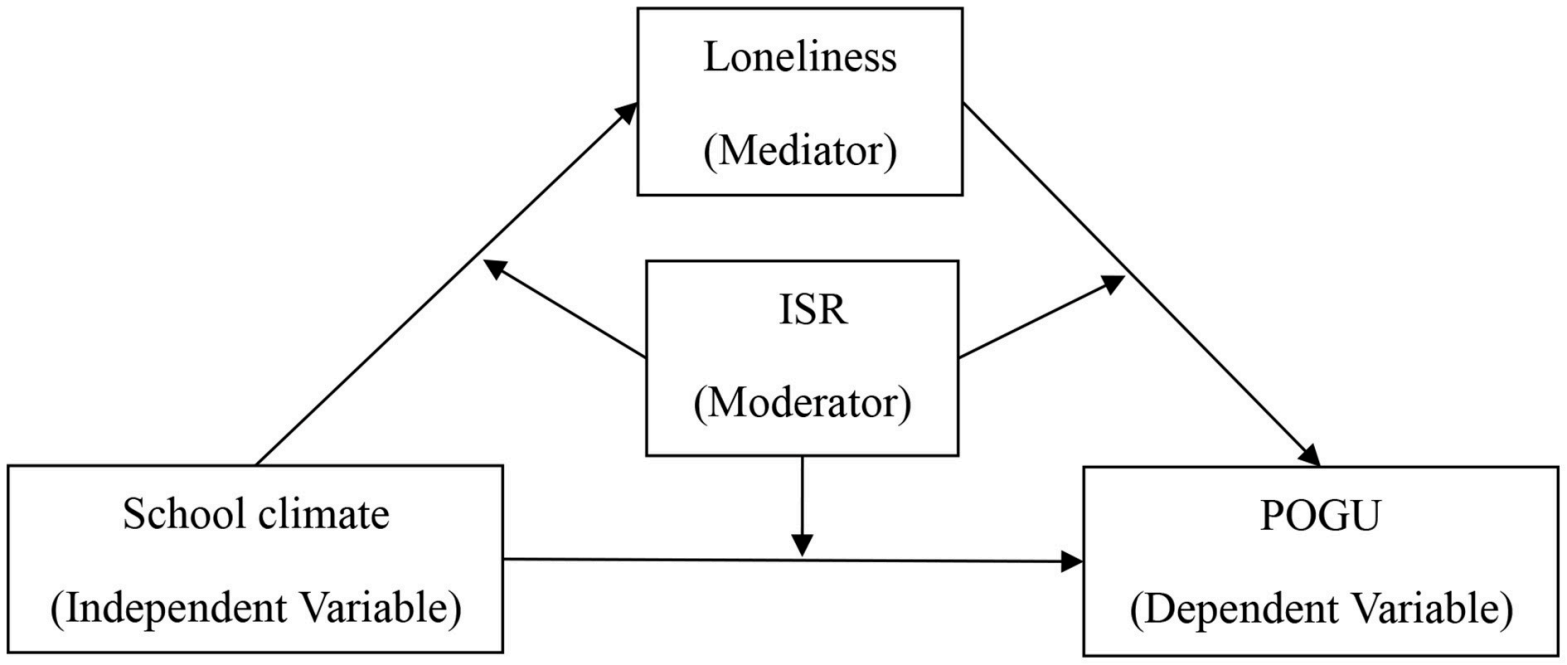
The proposed moderated mediation model. ISR, intentional self-regulation; POGU, problematic online game use.

Grounded in the self-system processes model and the ecological system theory, this study investigated whether loneliness mediates the relation between school climate and adolescent POGU and whether this indirect link is moderated by intentional self-regulation. Figure 1 illustrates the proposed research model.

## Method

### Participants

The participants in this study were recruited from two junior middle schools in Guangdong province, southern China, through stratified and random cluster sampling. A total of 500 adolescents (50.60% male) ranging in age from 12 to 17 (Mean_age_ = 13.59, *SD* = 0.65) participated in February 2019. Of those, 207 adolescents came from one school (Urban areas) while 293 came from the other (Rural areas). And Chi-square and *t*-tests showed that there were no differences between students from urban and rural areas.

### Procedure

We obtained written informed consent from both participants themselves and their parents before beginning all data collection. The data were collected in classrooms by well-trained psychology graduate students. Before the formal test, data collectors informed participants that participation was voluntary and that any uncomfortable questions need not be answered. Participants were also assured that their responses would be kept strictly confidential and that they would only be used for academic survey research. Adolescents received a pencil for their participation. In addition, our testing material and survey procedures were approved by the ethics in human research committee of School of Education, Guangzhou University, and School of Psychology, South China Normal University.

### Measures

Data were collected using School Climate Questionnaire, Intentional Self-regulation Questionnaire, Loneliness Scale, POGU Questionnaire, Parent-adolescent Relationship Questionnaire, and Impulsivity Scale.

#### School Climate

Adolescents reported perceived school climate using a 25-item version of a perceived school climate questionnaire (9). This questionnaire demonstrated good reliability and validity in Chinese adolescents (9, 18, 36). It assesses three dimensions: teacher-student support, student-student support, and opportunities for autonomy. Adolescents rated how often the statements applied to them on a 5-point scale ranging from 1 = never to 5 = always. The responses were averaged across the 25 items to form a composite score, with higher scores reflecting higher levels of positive school climate. For this study, the Cronbach's alpha was 0.86, which suggests that this questionnaire had fair internal consistency.

#### Intentional Self-Regulation

Adolescents reported their intentional self-regulation using a 9-item version of the intentional self-regulation questionnaire (29, 36). This questionnaire assesses three dimensions of intentional self-regulation: selection (e.g., “When I think about what I want in life, I commit myself to one or two important goals”), optimization (e.g., “When I want to achieve something difficult, I wait for the right moment and the best opportunity”), and compensation (e.g., “When things aren't going so well, I accept help from others”). Adolescents indicated how true each item was of them on a 5-point scale ranging from 1 = not at all true to 5 = very true. Responses across the nine items were averaged, with higher scores representing higher levels of intentional self-regulation. For this study, the Cronbach's alpha was 0.91, indicating that the scale had good internal consistency.

#### Loneliness

Adolescents reported their loneliness using the UCLA loneliness scale (37). This scale contains 20 items, which assess feelings of social isolation (e.g., “could not find companionship when I wanted it”). Participants rated the extent to which each statement applied to them on a 4-point scale ranging from 1 = not at all to 4 = always. Responses across the 20 items were averaged, with higher scores representing greater loneliness. For this study, the Cronbach's alpha was 0.90, which indicated that the scale had good internal consistency.

#### POGU

POGU was measured using the Chinese version Problematic Online Game Use Questionnaire (19). The instrument has demonstrated good reliability and validity in Chinese adolescent samples (19, 38, 39). Adolescents rated how often each statement (e.g., “Have you spent more time playing online games than was planned?”) was true for them on a 3-point scale: 0 = never, 1 = sometimes, and 2 = yes. The answers were recoded into “never” = 0, “sometimes” = 0.5, and “yes” = 1. This mode of scoring is more accurate because it allows participants who “sometimes” experienced symptoms to be considered (19, 40). The grand total score of the 11 items was calculated, with higher scores representing greater severity of POGU. For this study, the Cronbach's alpha was 0.89, which indicated that the questionnaire had good internal consistency.

#### Control Variables

Given that prior studies shown that adolescents' gender, age, parent-adolescent relationship, and impulsivity were associated with POGU (40–42), we include them as control variables in statistical models. Parent-adolescent relationship was assessed using the Chinese version Parent-adolescent Relationship Questionnaire (43), and impulsivity was assessed using the Urgency-Premeditation-Perseverance-Sensation seeking-Positive Urgency (UPPS-P) Scale (44). For this study, father-adolescent relationship, mother-adolescent relationship, and impulsivity all demonstrated excellent internal consistency (Cronbach's α are 0.78, 0.78, and 0.86 respectively).

### Statistical Analyses

Descriptive statistics were conducted via use of SPSS 25.0. And Mplus 7.1 was utilized to examine mediation and moderation effects by conducting structural equation modeling analysis (45).

## Results

### Prevalence of POGU

According to the opinions of POGU experts (4, 40), adolescents who exhibited at least 5 of the 11 criteria on the POGU questionnaire were considered to be addict gamers. In the current sample, 5.40% of the participants displayed signs of gaming addiction. This rate is consistent with national Chinese adolescent data (8) and recent literature (19).

### Preliminary Analyses

The means, standard deviations, and correlation coefficients for all variables of the current study are displayed in Table 1. The results showed that school climate and intentional self-regulation were both negatively related to loneliness and POGU, whereas loneliness was positively related to POGU. These findings suggest that a negative school climate, low intentional self-regulation, and high loneliness all were potential risk factors for POGU, and a negative school climate and low intentional self-regulation were both potential risk factors for loneliness.

**Table 1:** Descriptive statistics and correlations for all variables.

**Variables**	**1**	**2**	**3**	**4**	**5**	**6**	**7**	**8**	**9**	**10**
1.Gender	1.00									
2.Age	0.08	1.00								
3.Area	−0.09	−0.20^**^	1.00							
4.FAR	0.09^*^	−0.05	−0.02	1.00						
5.MAR	0.10^*^	−0.03	−0.06	0.68^**^	1.00					
6.Impulsivity	0.01	−0.05	0.05	−0.28^**^	−0.34^**^	1.00				
7.School climate	−0.16^**^	−0.13^**^	−0.08	0.21^**^	0.22^**^	−0.31^**^	1.00			
8.ISR	0.04	−0.02	−0.08	0.18^**^	0.21^**^	−0.42^**^	0.32^**^	1.00		
9.Loneliness	0.01	−0.03	0.09	−0.23^**^	−0.25^**^	0.36^**^	−0.33^**^	−0.35^**^	1.00	
10.POGU	0.31^**^	0.04	−0.01	−0.13^**^	−0.18^**^	0.26^**^	−0.26^**^	−0.18^**^	0.24^**^	1.00
*M*	0.51	13.59	0.41	2.44	2.51	2.21	3.18	3.48	2.01	1.26
*SD*	0.50	0.55	0.49	0.37	0.36	0.40	0.38	0.62	0.51	1.96

*Gender and area were dummy coded such that 1 = male, 0 = female, and 1 = urban, 0 = rural. FAR, father-adolescent relationship; MAR, mother-adolescent relationship; ISR, intentional self-regulation; POGU, problematic online game use*.

* *p < 0.05*,

** *p < 0.01*.

### Testing the Moderating Effect of Intentional Self-Regulation on the Direct Link Between School Climate and Adolescent POGU

**Figure 2:**
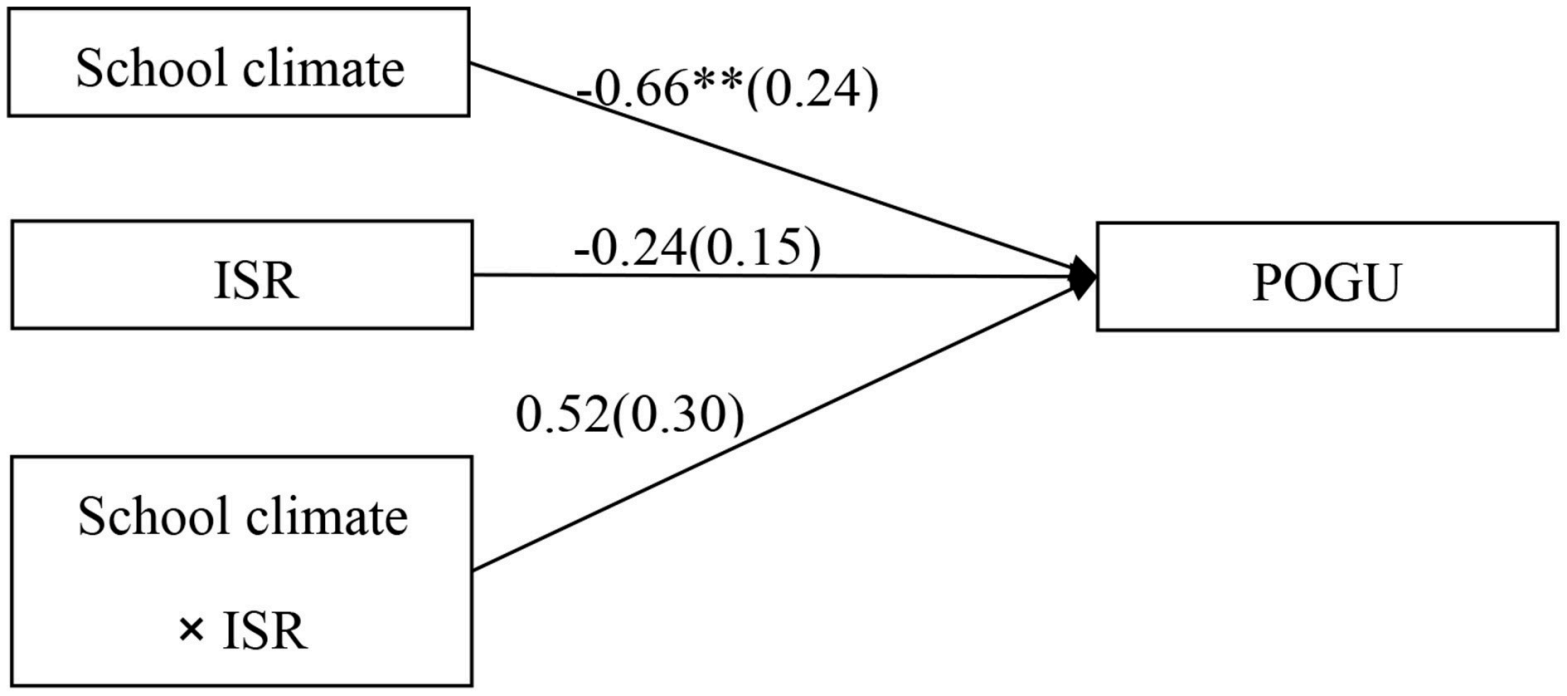
Model of the moderating role of intentional self-regulation on the direct relationship between school climate and POGU. ISR, intentional self-regulation; POGU, problematic online game use. Values are unstandardized coefficients and standard error. Paths between gender, age, father-adolescent relationship, mother-adolescent relationship, impulsivity, and each of the variables in the model are not displayed. Of those paths, the following were significant: gender (*b* = 1.16, *SE* = 0.16, *t* = 7.12^**^), and impulsivity (*b* = 0.78, *SE* = 0.23, *t* = 3.35^**^) to POGU. ^**^p < 0.01.

The moderated model which was shown in Figure 2 revealed an acceptable fit to the data: χ^2^/*df* = 4.29, CFI = 0.91, RMSEA = 0.069. The results demonstrated that the main effects of school climate was significantly associated with POGU (*b* = −0.66, *SE* = 0.24, *t* = −2.77, *p* < 0.01), however, the main effects of intentional self-regulation (*b* = −0.24, *SE* = 0.15, *t* = −1.64, *p* > 0.05), and the interactive effect of school climate and intentional self-regulation (*b* = 0.52, *SE* = 0.30, *t* = −1.75, *p* > 0.05) were non-significantly associated with POGU.

### Testing for Mediation Effect of Loneliness

**Figure 3:**
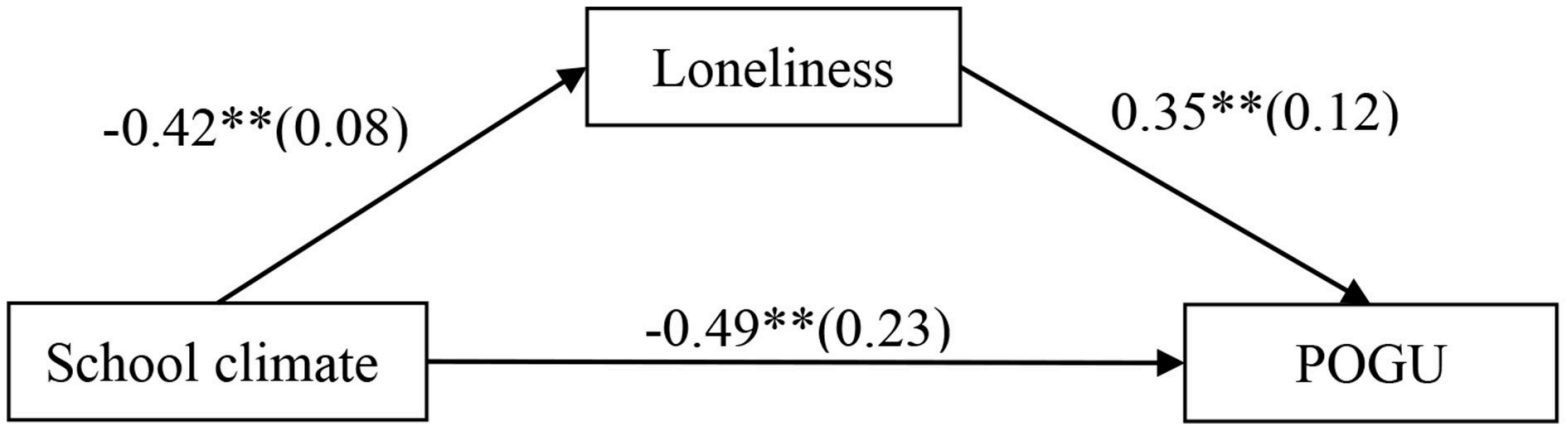
Model of the mediating role of loneliness between school climate and POGU. POGU, problematic online game use. Values are unstandardized coefficients and standard error. Paths between gender, age, father-adolescent relationship, mother-adolescent relationship, impulsivity, and each of the variables in the model are not displayed. Of those paths, the following were significant: impulsivity to loneliness (*b* = 0.44, *SE* = 0.08, *t* = 5.49^**^); gender (*b* = 1.16, *SE* = 0.16, *t* = 7.19^**^), and impulsivity (*b* = 0.74, *SE* = 0.23, *t* = 3.29^**^) to POGU. ^**^p < 0.01.

The mediation model represented in Figure 3 revealed an excellent fit to the data: χ^2^/*df* = 2.39, CFI = 0.96, RMSEA = 0.033. The results are displayed in Figure 3. School climate negatively predicted loneliness (*b* = −0.42, *SE* = 0.08, *t* = −5.13, *p* < 0.01) and negatively predicted POGU (*b* = −0.49, *SE* = 0.23, *t* = −2.12, *p* < 0.05), and loneliness positively predicted POGU (*b* = 0.35, *SE* = 0.12, *t* = 2.86, *p* < 0.01). Moreover, bootstrapping analyses indicated that loneliness partially mediated the relation between school climate and adolescent POGU (indirect effect = −0.1482, *SE* = 0.0676, 95% CI = [−0.3100, −0.0353]).

### Testing for Moderated Mediation

**Figure 4:**
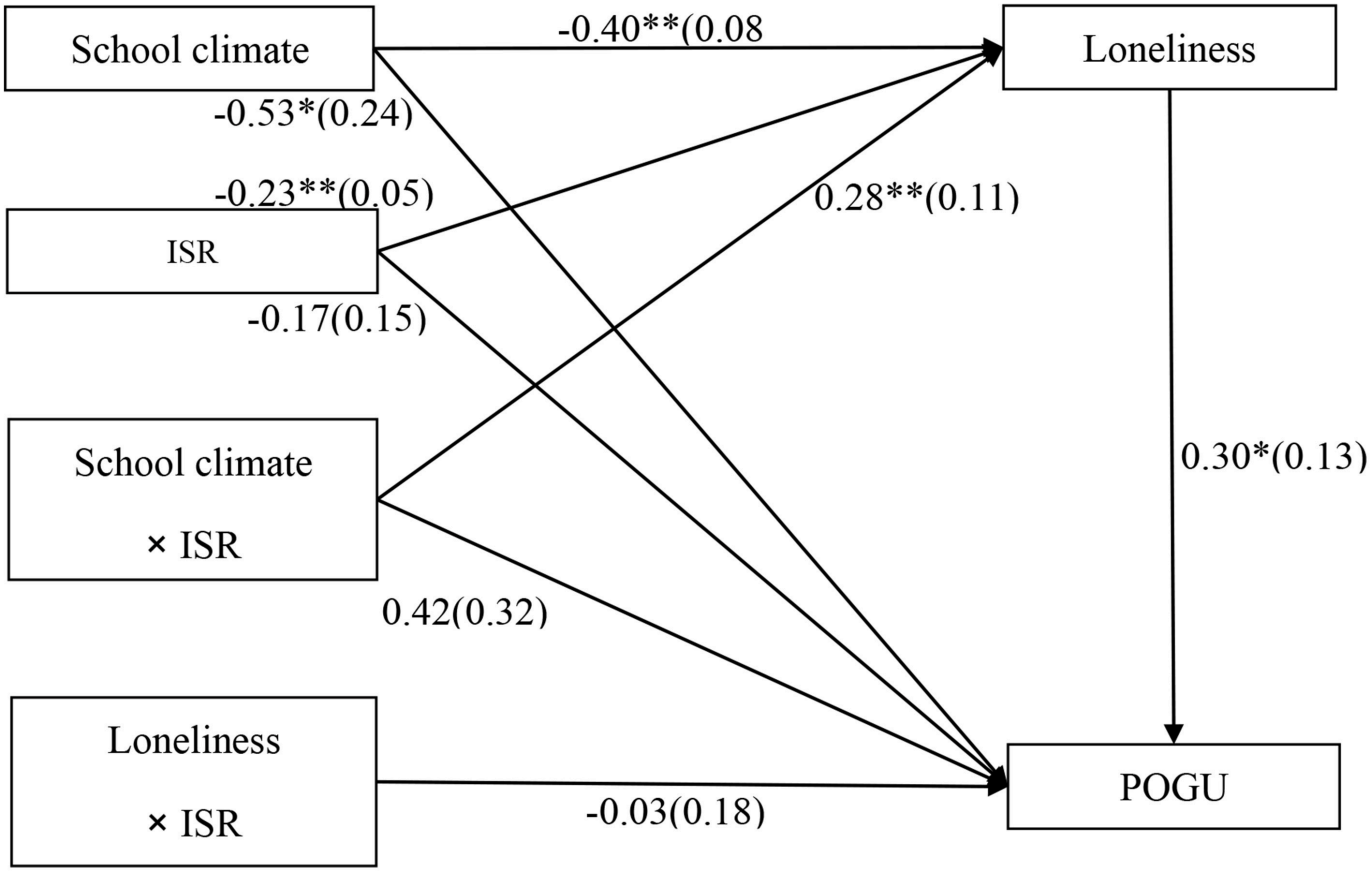
Model of the moderating role of intentional self-regulation on the indirect relationship between school climate and POGU. ISR, intentional self-regulation; POGU, problematic online game use. Values are unstandardized coefficients and standard error. Paths between gender, age, father-adolescent relationship, mother-adolescent relationship, impulsivity, and each of the variables in the model are not displayed. Of those paths, the following were significant: impulsivity to loneliness (*b* = 0.33, *SE* = 0.08, *t* = 3.91^**^); gender (*b* = 1.16, *SE* = 0.16, *t* = 7.15^**^), and impulsivity (*b* = 0.69, *SE* = 0.24, *t* = 2.89^**^) to POGU. ^*^p < 0.05, ^**^p < 0.01.

**Figure 5:**
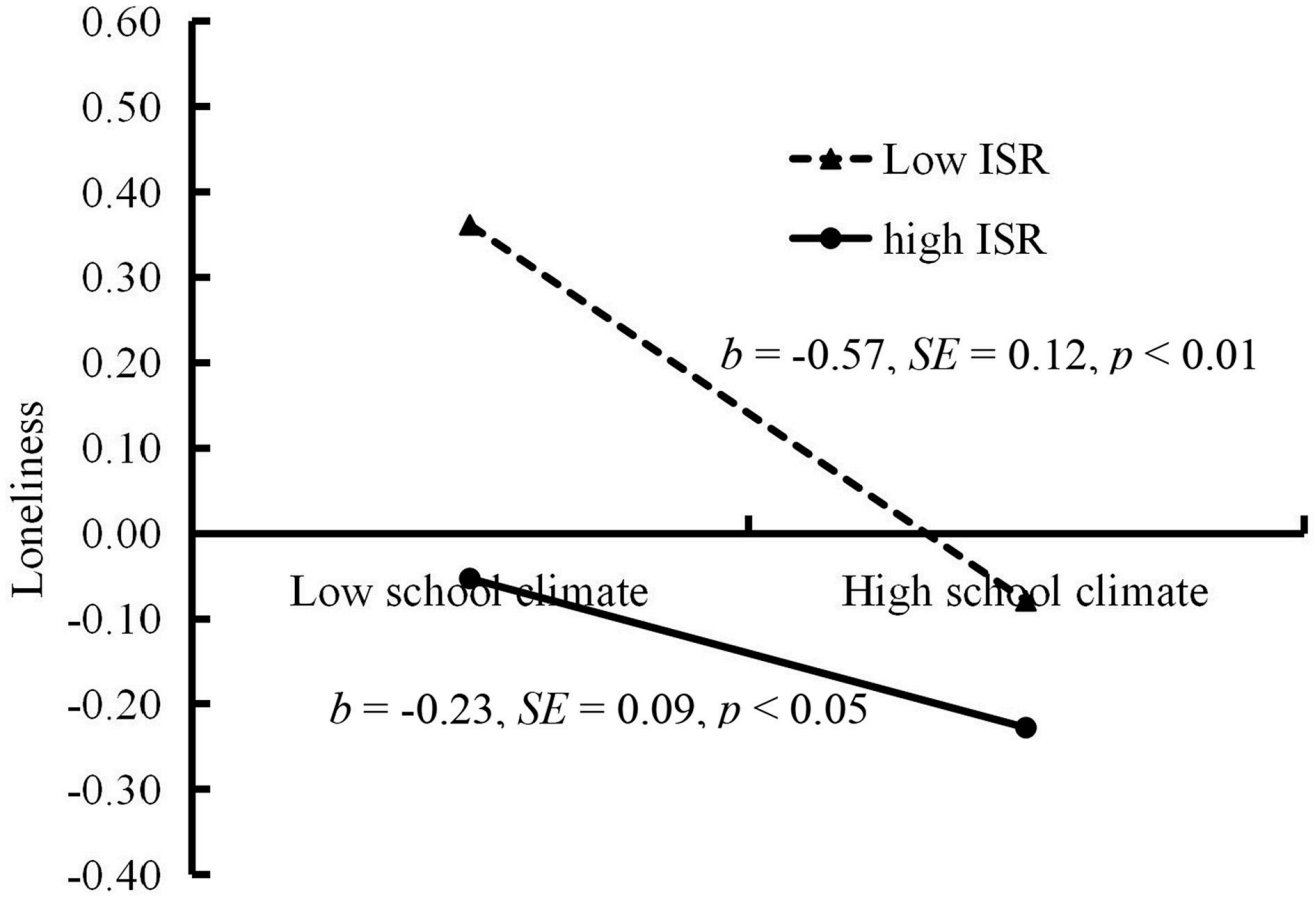
Loneliness among adolescents as a function of school climate and intentional self-regulation. ISR, intentional self-regulation.

The moderated mediation model represented in Figure 4 revealed a good fit to the data: χ^2^/*df* = 3.16, CFI = 0.92, RMSEA = 0.043. The bias-corrected percentile bootstrap results indicated that the indirect effect of school climate on adolescent POGU through loneliness was moderated by intentional self-regulation. Specifically, intentional self-regulation moderated the association between school climate and loneliness (*b* = 0.28, *SE* = 0.11, *t* = 2.64, *p* < 0.01). We conducted a simple slopes test, and as depicted in Figure 5, the negative link between school climate and loneliness was much stronger for adolescents with lower intentional self-regulation (1*SD* below the mean; *b* = −0.57, *SE* = 0.12, *t* = −4.85, *p* < 0.01) than for adolescents with higher intentional self-regulation (1*SD* above the mean; *b* = −0.23, *SE* = 0.09, *t* = −2.40, *p* < 0.05). Moreover, school climate was negatively associated with loneliness (*b* = −0.40, *SE* = 0.08, *t* = −4.73, *p* < 0.01) and POGU (*b* = −0.53, *SE* = 0.24, *t* = −2.21, *p* < 0.05). However, the interaction between intentional self-regulation and loneliness in predicting adolescent POGU was no significant (*b* = −0.03, *SE* = 0.18, *t* = −0.15, *p* > 0.05).

Moreover, the indirect link between school climate and POGU via loneliness were significant for adolescents with lower intentional self-regulation (indirect effect = −0.17, *SE* = 0.07, 95% CI [−0.32, −0.02]) and for those with higher intentional self-regulation (indirect effect = −0.07, *SE* = 0.04, 95% CI = [−0.13, −0.01]). Adolescents with lower intentional self-regulation were more likely to develop loneliness, which in turn contributed to higher levels of POGU.

## Discussion

The first goal of this study was to explore the mediating effect of loneliness on the relationship between school climate and adolescent POGU. Consistent with our hypothesis 1, this study found that loneliness significantly mediated the effect of school climate on adolescent POGU. Previous research has demonstrated that the school climate is associated with loneliness (23–25) and that the latter is associated with increased risk of POGU (5, 6, 26). We integrated these two links in the current study with a mediation modeling approach. The findings of this study suggest that loneliness is an essential underlying psychosocial process that helps explain why a favorable school climate is linked with less POGU and why a negative school climate is linked with more POGU. When adolescents have positive experiences, perceive more support from their teachers and peers, and report more autonomy, they are less likely to feel lonely, which in turn is associated with less POGU.

This finding is in line with the self-system processes model (21). It is also congruent with previous research showing that the protective effects of social context on adolescent developmental outcomes (i.e., school climate, social support, family climate) are mediated by psychological processes including loneliness (24, 46, 47). According to self-determination theory (48), contextual factors (i.e., school climate) influence adolescent behaviors (i.e., POGU) through the mediating effects of internal psychology. More concretely, when a school climate cannot satisfy an adolescent's need for autonomy and relatedness, he or she feels lonely and thus seeks an environment through which he/she can meet his/her psychological needs and reduce feelings of loneliness. Online games offer a setting in which people can express themselves in ways that they may not feel comfortable doing in real life, and it can also be a good place for people to make new friends and socialize. Survey research has indicated that players may gain a sense of belonging from an online game and that social communication and relationships are important motivators for engagement in online games (49). In contrast, when a school climate promotes positive emotional student-teacher and student-student bonds, students may not feel lonely at school. Thus, students tend to make efforts to control their behavior so that their actions will be in accordance with social expectations and are therefore less likely to become addicted to online games. Therefore, a positive school climate may be effective in treating loneliness, which may be a promising approach for adolescent POGU prevention and cessation.

The second goal of this study was to explore the moderating effect of intentional self-regulation on the indirect association between school climate and POGU via loneliness. Consistent with the risk-buffering hypothesis and with our own hypothesis, this study found that intentional self-regulation weakened the link between school climate, loneliness, and POGU through the direct relationship between school climate and loneliness. Specifically, the negative association between school climate and loneliness was stronger among adolescents with low intentional self-regulation, which in turn increased their POGU. This is because adolescents with higher intentional self-regulation tend to have more resources and greater capacity to select appropriate goals, apply and refine relevant means of achieving positive outcomes, and avoid losses (32). This pattern of moderator effects has also been found in the association between environmental factors (i.e., school climate, family environment) and externalizing behaviors. For example, Lin et al. (50) reported that the negative link between school climate and adolescent smoking behavior via deviant peer affiliation was substantially stronger among adolescents with lower intentional self-regulation than among those with higher intentional self-regulation. Similarly, Yuan (51) found that among adolescents with low intentional self-regulation, parental corporal punishment could have increased their deviant peer affiliation, which in turn increased POGU. In contrast, among adolescents with high intentional self-regulation, the relation was not significant. Although these studies have found that intentional self-regulation diminishes the indirect link between environmental factors and adolescent development, they used externalizing behaviors such as deviant peer affiliation as mediating variables rather than loneliness (50, 51). Therefore, this research extended the range of the moderating effect of intentional self-regulation to internalizing behaviors by loneliness.

This study also examined whether the relationship between loneliness and adolescent POGU was moderated by intentional self-regulation. The findings showed that this moderating effect was non-significant. These findings suggest that intentional self-regulation can help to promote a positive school climate and reduce adolescent loneliness, which in turn can reduce the risk of POGU. However, intentional self-regulation cannot eliminate the risk of adolescent POGU merely due to its effects on loneliness. Even so, this study contributes to the literature by enhancing our understanding of adolescent POGU etiology and suggesting the potential success of improving intentional self-regulation as a personal capability in POGU intervention programs.

### Practical Implications

The findings of this study have important theoretical and practical implications. Our findings suggest that loneliness is an important mediator in the relation between perceived school climate and POGU. Thus, teachers and parents may prevent adolescent POGU and intervene in this behavior by reducing adolescents' loneliness. Moreover, our findings suggest that the negative link between school climate and adolescent POGU through loneliness is stronger for adolescents with poor intentional self-regulation than for those with high intentional self-regulation. Therefore, it is important to foster more positive perceptions of school climate among adolescents, especially among those with poor intentional self-regulation.

### Limitations

Several limitations should be noted regarding this study. First, the data were collected using self-report measures; thus, common method biases may have existed. Second, this study only adjusted for the covariates of adolescents' gender, age, parent-adolescent relationship, and impulsivity. Future research should consider other relevant control variables, such as family function and peer context. Third, as the generalization of our results from this small sample of Chinese adolescents was difficult, future research should attempt to recruit larger samples from wider cultural and/or geographical settings for the purpose of clarifying the relationships between the variables in this study.

## Ethics Statement

Testing material and survey procedures were approved by the ethics in human research committee of School of Education, Guangzhou University, and School of Psychology, South China Normal University.

## Author Contributions

CY, WL, XL, and WZ designed the work. CY, QL, HL, and WZ collected the data. CY, QL, HL, WL, XL, and WZ analyzed the data results and drafted the manuscript. CY, QL, WZ, HL, KD, XX, XG, WL, and XL revised the manuscript.

### Conflict of Interest Statement

The authors declare that the research was conducted in the absence of any commercial or financial relationships that could be construed as a potential conflict of interest.
